# Polymorphism in autophagy-related genes *LRP1* and *CAPZA1* may promote gastric mucosal atrophy

**DOI:** 10.1186/s41021-023-00274-5

**Published:** 2023-05-17

**Authors:** Naoyuki Yamaguchi, Takuki Sakaguchi, Hajime Isomoto, Tatsuo Inamine, Ryoya Tsukamoto, Daisuke Fukuda, Ken Ohnita, Tsutomu Kanda, Kayoko Matsushima, Tatsuro Hirayama, Kazuo Yashima, Kazuhiro Tsukamoto

**Affiliations:** 1 Department of Gastroenterology and Hepatology, Nagasaki University Graduate School of Biological Sciences, 1-7-1 Sakamoto, Nagasaki, 852-8501 Japan; 2 Department of Gastroenterology and Nephrology, Faculty of Medicine, Tottori University, 36-1 Nishi-Cho, Yonago, 683-8504 Japan; 3 Department of Pharmacotherapeutics, Nagasaki University Graduate School of Biomedical Sciences, 1-7-1 Sakamoto, Nagasaki, 852-8501 Japan; 4 Department of Surgical Oncology, Nagasaki University Graduate School of Biological Sciences, 1-7-1 Sakamoto, Nagasaki, 852-8501 Japan; 5 Fukuda Yutaka Clinic, 3-5 Hamaguchi-machi, Nagasaki, 852-8107 Japan; 6 Shunkaikai Inoue Hospital, 6-12 Takara-machi, Nagasaki, 850-0045 Japan

**Keywords:** Cytotoxin-associated gene A, Gastric mucosal atrophy, Autophagy-related genes, *LRP1*, *CAPZA1*, *LAMP1*, SNP

## Abstract

**Background:**

*Helicobacter pylori* secretes cytotoxin-associated gene A (CagA) into the gastric epithelium, causing gastric mucosal atrophy (GMA) and gastric cancer. In contrast, host cells degrade CagA via autophagy. However, the association between polymorphisms in autophagy-related genes and GMA must be fully elucidated.

**Results:**

We evaluated the association between single nucleotide polymorphisms (SNPs) in autophagy-related genes (low-density lipoprotein receptor-related protein 1, *LRP1*; capping actin protein of muscle Z-line alpha subunit 1, *CAPAZ1*; and lysosomal-associated membrane protein 1, *LAMP1*) and GMA in 200 *H. pylori*-positive individuals. The frequency of the T/T genotype at rs1800137 in *LRP1* was significantly lower in the GMA group than in the non-GMA group (*p* = 0.018, odds ratio [OR] = 0.188). The frequencies of the G/A or A/A genotype at rs4423118 and T/A or A/A genotype at rs58618380 of *CAPAZ1* in the GMA group were significantly higher than those in the non-GMA group (*p* = 0.029 and *p* = 0.027, respectively). Multivariate analysis revealed that C/C or C/T genotype at rs1800137, T/A or A/A genotype at rs58618380, and age were independent risk factors for GMA (*p* = 0.038, *p* = 0.023, and *p* = 0.006, respectively). Furthermore, individuals with the rs1800137 C/C or C/T genotype of *LRP1* had a 5.3-fold higher susceptibility to GMA. These genetic tests may provide future directions for precision medicine for individuals more likely to develop GMA.

**Conclusion:**

*LRP1* and *CAPZA1* polymorphisms may be associated with the development of GMA.

## Introduction

*Helicobacter pylori*, a gram-negative bacillus first isolated from human gastric mucosa in 1983 [1, 2], is defined as a Group I carcinogen by the International Agency for Research on Cancer (IARC) [3]. Approximately half of the world’s population is estimated to be infected with *H. pylori* [4, 5]. Chronic *H. pylori* infection causes chronic gastritis, which progresses to atrophic gastritis, intestinal metaplasia, and finally to gastric cancer, as described by Correa’s cascade [6]. Epidemiological data have demonstrated that *H. pylori* infection increases the risk of noncardiac gastric cancer by approximately 3-fold. However, not all infected patients develop gastric cancer or severe atrophic gastritis. Approximately 10–20% of *H. pylori*-infected individuals develop gastrointestinal diseases [7], and 1–3% of infected individuals develop gastric carcinoma (GC) [8, 9]. The risk of developing gastric cancer increases with the degree of gastric mucosal atrophy (GMA) [10]. Therefore, preventing the progression of GMA is essential.

GMA development has been attributed to both pathogen- and host-specific factors. The most well-known pathogenic factor of *H. pylori* is cytotoxin-associated gene A (CagA). Upon infection of the epithelial mucosa, *H. pylori* injects CagA, a virulence factor associated with increased gastric cancer risk, into the host cell cytosol using the Cag type IV secretion system (T4SS) [11]. Once injected into the host cytosol, CagA exerts multiple effects on the epithelial cells and induces several histopathological changes in the gastric epithelium [12]. Moreover, CagA induces inflammatory cytokines, such as interleukin (IL)-1, IL-6, IL-18, tumor necrosis factor α (TNFα), and interferon-γ (IFN-γ), which activate NF-κB [13–15] and prolong inflammation. Therefore, injected CagA is associated with atrophic gastritis [3].

**Fig. 1:**
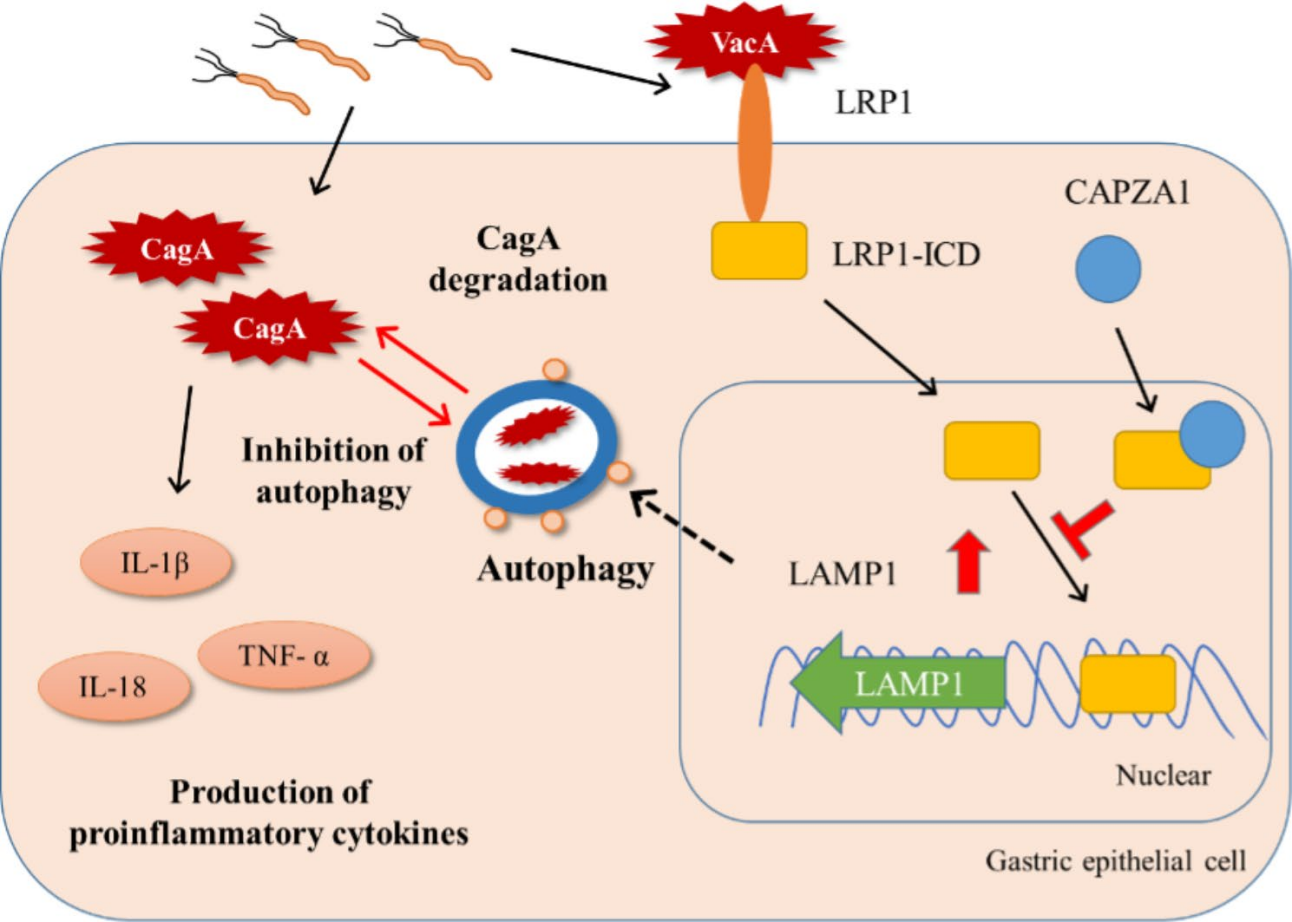
Putative model of autophagy as a host defense against *H. pylori* infection. *H. pylori* injects the toxic protein, CagA, into host epithelial cells. Then, CagA induces the production of pro-inflammatory cytokines. The host cell recognizes the VacA toxin secreted by *H. pylori* via the *LRP1* receptor and induces autophagy. LRP1-ICD moves into the nucleus and increases the expression of LAMP1. Subsequently, autophagy degrades CagA. However, CAPZA1, a host protein, binds to the LRP1-ICD and inhibits LRP1-induced autophagy

CagA is degraded by autophagy, a system for bulk protein degradation and elimination of invading pathogens (Fig. 1). In autophagy systems, the isolation membrane segregates a small portion of the cytoplasm, soluble materials, and organelles into an autophagosome structure. The autophagosome fuses with the autolysosome, and its contents are degraded [16]. Autophagy is induced by vacuolating cytotoxin A (VacA) secreted by *H. pylori*. VacA binds to low-density lipoprotein receptor-related protein 1 (*LRP1)* [17]. Then, the intracellular domain of LRP1 (LRP-ICD) migrates to the nucleus and binds to the promoter of lysosomal-associated membrane protein 1 (LAMP1), increasing LAMP1 expression. LAMP1, abundant in lysosomal membranes, is required for autolysosome formation [18]. Thus, VacA reduces intracellular CagA levels by inducing autophagy [17, 19]. In contrast, the capping actin protein of the muscle Z-line alpha subunit 1 (*CAPZA1*) acts as a negative regulator by binding to LRP-ICD [20].

Recently, Nakamura et al. reported that LRP1 mutations increase CagA accumulation in non-invasive gastric cancer [21]. Furthermore, Tsugawa et al. reported that CAPZA1 overexpression reduced CagA degradation [20]. However, the relationship between gene polymorphisms related to LAMP1 expression and GMA progression has not yet been elucidated. Here, we performed an association study between single nucleotide polymorphisms (SNPs) of autophagy-related genes (*LRP1, LAMP1*, and *CAPZA1*) and GMA progression.

## Materials and methods

### Subjects

Of the 503 individuals who underwent esophagogastroduodenoscopy for a health checkup at Fukuda Surgical Hospital (Nagasaki, Japan) between August and December 2013, 200 with *H. pylori* antibody titers ≥ 10 U/mL (E-plate Eiken *H. pylori* antibody II; Eiken Chemical, Tokyo, Japan) were enrolled in this study. The inclusion criteria were age < 80 years and no eradication history of *H. pylori*. Written informed consent for genetic analysis was obtained from all participants, and blood samples were collected. This study was approved by the Ethical Review Board of Human Genome Gene Analysis Research, Nagasaki University (No. 120,221, approved on February 16th, 2012).

### GMA classification

Pepsinogen assays were used to classify participants as GMA-positive or -negative. Participants with pepsinogen (PG) I levels ≤ 70 µg/L (PG I ≤ 70) and PG I/II ratio ≤ 3.0 (PG I/II ≤ 3.0) were classified into the GMA group. The remaining participants were classified into the non-GMA group [22, 23].

### Endoscopic GMA classification

The Kimura–Takemoto classification [24] was used to classify subjects as endoscopic-GMA-positive (EGMA) or endoscopic-GMA-negative (non-EGMA). Participants with C-III or open type were classified into the EGMA group. The remaining participants were classified into the non-EGMA group.

### Extraction of DNA from peripheral blood

DNA was extracted from the peripheral blood of participants using NucleoSpin Blood DNA extraction kits (Takara Bio, Shiga, Japan). The concentration of the extracted DNA was measured using a Nanodrop 1000 spectrophotometer (Nanodrop Technologies, Wilmington, DE, USA) and diluted to 10 ng/µL with low TE buffer (10 mM Tris-HCl, pH 8.0, 0.1 mM EDTA).

### Selection of tag SNPs in candidate genes

**Fig. 2:**
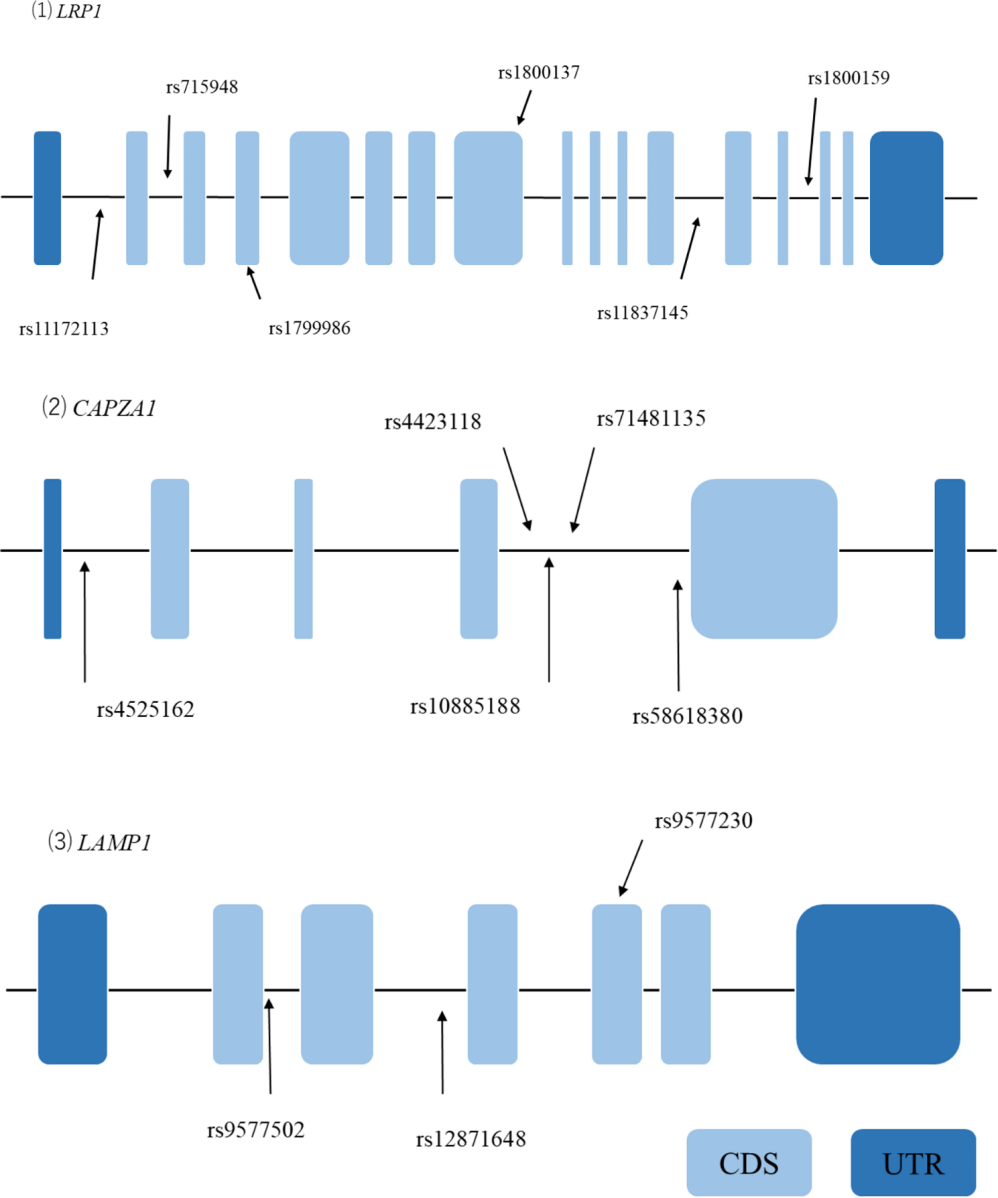
Gene structures and locations of genotyped tag SNPs in *LRP1*, *CAPAZ1*, and *LAMP1*. Horizontal lines indicate the base sequence of each gene. Boxes represent exons. Arrows indicate the positions of the genotyped tag SNPs. Abbreviations: CDS, coding sequence; UTR, untranslated region

Using the 1000 Genome Project (GRCh37 p.13) database, all SNPs in the *LRP1* (NCBI Gene ID: 4035) region and its 3 kb upstream promoter region in Japanese individuals were extracted. SNPs with minor allelic frequencies > 0.1 were selected from the extracted SNPs. Tag SNPs were selected by a pair-wise tagging method (r^2^ > 0.8) using Haploview software (version 4.2) [25]. Tag SNPs of *CAPZA1* (ID: 829) and *LAMP1* (ID: 3916) were selected in the same way as the *LRP1* gene. The positions of the tag SNPs in the three genes are shown in Fig. 2.

### Genotyping

The selected tag SNPs were genotyped as follows: primers for polymerase chain reaction (PCR) were designed to contain each tag SNP; primer sequences, annealing temperatures, and cycle numbers used in these reactions are listed in Table 1.

**Table 1:** Characteristics of tag SNP genotyping primers, anneaking temperature, and cycle numbers

Gene	SNP	Major > Minor		Sequence of primer pairs (5' to 3’)	Annealing temperature(℃)	Cycle numbers
*LRP1*	rs715948	T > C	Forward	TCCCTTTCCAGCCCTAAAAT	64.0	50
			Reverse	GCCCCCAGTAAGTGGTGTAA		
	rs1799986	C > T	Forward	CACCATAGCCAGCTTGTTCA	62.5	50
			Reverse	CGGAAGGTGGGCTGATAATA		
	rs1800137	C > T	Forward	AAGCTCGTCGACAGCAAGAT	63.5	50
			Reverse	GTTCCCCTACAGAGGCTTCC		
	rs1800159	G > A	Forward	GCTAGCCAGGTGAGGCTGT	66.0	50
			Reverse	GGTGTGGAGGTGTCTGTGTG		
	rs11172113	T > C	Forward	ACTCAGAATGGAAGCGGAGA	61.0	50
			Reverse	AGAGCCTGCAGGAATCTGAA		
	rs11837145	C > A	Forward	CACAGGTCCCATCCAAGTG	62.5	50
			Reverse	CTCCAGGCCATTCAAAGGTA		
*CAPZA1*	rs4423118	G > A	Forward	GATCAGAAAGGCAGCGACTC	62.0	50
			Reverse	CTGCAAGTTTTACCCCAAGG		
	rs4525162	C > T	Forward	CTCCCCTGGGAGGTCATTAT	62.5	50
			Reverse	ACCTCTGTTGGGTGAAGTGG		
	rs10885188	G > A	Forward	GTGTTGCTTCTATGTGCCTTTG	62.0	50
			Reverse	AACAGCCTTCTGCAGCAAAT		
	rs58618380	T > A	Forward	AAGCTCTGTTTGGAGGCAAG	61.0	50
			Reverse	GAGCTTCCTTTGCCAGTTTG		
	rs71481135	T > C	Forward	GGAGTGCAATGTCTGCTGAG	62.5	50
			Reverse	CCTCACCCATGATAGGCAGT		
*LAMP1*	rs9577230	T > C	Forward	TGCAAGTTCTAGCCGGTTTT	61.0	50
			Reverse	AGGGTCTGCAACACACACAG		
	rs9577502	A > T	Forward	GACATGCAATGCAAACACTG	62.0	50
			Reverse	TTTGGAGATGGTCTCGCTCT		
	rs12871648	A > C	Forward	AGCCAGGCTTTTGGGTTTAT	65.6	50
			Reverse	CAAATTGGGTGGGAGATGAG		

### PCR-high resolution melting curve analysis with unlabeled probe

Each polymorphic region was amplified by PCR using a GeneAmp PCR 9700 system (Life Technologies, Carlsbad, CA, USA) or T100 Thermal Cycler (Bio-Rad, Hercules, CA, USA). Amplification reactions were performed in 15 µL total volume containing 10 ng of genomic DNA, 0.06 µM forward primer, 0.3 µM reverse primer, 5X Colorless Go Taq Flexi Buffer (Promega, Madison, WI, USA), 25 mM MgCl_2_ solution, 10 mM each PCR Nucleotide Mix, 0.3 µM Go Taq G2 Hot Start Polymerase (Promega), 0.3 µM probe, and 0.6 µM SYTO9 (Life Technologies) (Table 2). The probes were oligonucleotides of 25–35 bases complementary to the major allele of the tag SNPs. The probes used for *LRP1* had an additional mismatch base at the 3′-end to prevent probe elongation.

**Table 2:** Probe sequence and melting temperatures of probe-based HRM

Gene	SNP	Genotyping	Probe-based HRM	
			Probe sequence (5‘to 3’)	Melting temperature (℃)
*LRP1*	rs715948	Probe-based HRM	AGGGGAGAGGCTAAATGTGGAGCCACCATAT	
	rs1799986	Probe-based HRM	AGGACTGCATGGACGGCTCAGATGAGATT	50→95
	rs1800137	Probe-based HRM	CCGCCAGACCATCATCCAGGGCATCGCA	50→95
	rs1800159	Probe-based HRM	AACACCTGCTCTGTCCTAGTGTCCTCATGCGAA	50→95
	rs11172113	Probe-based HRM	AGGAAAGAGCCACTGGGCAACACCCAAAATAGTT	50→95
	rs11837145	Probe-based HRM	CCCCACACTTCTGTTCATTGGGTTAGATTTTACA	50→95
*CAPZA1*	rs4423118	Probe-based HRM	TCACCTCCACATGCTGAGGTCAATACTGGATATC	50→95
	rs4525162	Probe-based HRM	CATTACATTTATGCTTCCAAGGAATCAATTGTCGCA	50→95
	rs10885188	Probe-based HRM	TCTCCTGAGAGAGACGCAATGAGAAGTTTATCGC	50→95
	rs58618380	Probe-based HRM	TGGCTGAAAGAACAATTGTTTTCCAGTTTCACGA	50→95
	rs71481135	Probe-based HRM	AAATAAAGCAATTCCTCATGCTGCCATGGCCGGC	50→95
*LAMP1*	rs9577230	Probe-based HRM	TCCAGTTGAATACAATTCTTCCTGACGCCAAGC	50→95
	rs9577502	Probe-based HRM	GACATAGTAAGATCTTATCTCTTTTTAAAAAAAAAAAAAATAC	50→95
	rs12871648	Probe-based HRM	TTGTTGAATGAATCAGAGGACTGCCTGACTCGAT	50→95

The probe sequence and melting temperature for probe-based HRM are shown.

Each PCR product was analyzed by high-resolution melting (HRM) curve using a LightCycler 480 instrument (Roche Diagnostics, Basel, Switzerland). HRM analysis consisted of heat denaturation at 95 °C for 1 min and re-annealing at 40 °C for 1 min. The melting curve of the probes was acquired by increasing the temperature and then analyzed using LightCycler 480 Gene Scanning software (version 1.5) to determine the genotype. Because the probes were complementary to the major alleles, probes with high-temperature melting curves were designated as homozygous for the major allele, probes with low-temperature melting curves were designated as homozygous for the minor allele, and probes with both melting curves were designated as heterozygotes. Arbitrarily selected samples were analyzed using PCR-direct DNA sequencing to determine probe accuracy.

### PCR-direct DNA sequencing

Each polymorphic region was amplified by PCR using the same reaction mixture and conditions described in the ‘Genotyping’ section above. The PCR product (5 µL) was reacted with Exonuclease I (Epicentre, Madison, WI, USA) and shrimp alkaline phosphatase (Affymetrix, Inc., CA, USA) to inactivate the PCR primers. Cycle sequencing reactions were performed using the BigDye Terminator cycle sequencing kit (version 3.1; Life Technologies) according to the manufacturer’s protocol. The reaction solution consisted of 25 ng of template DNA, sequencing buffer, 0.1 µM of forward primer or 0.1 µM of reverse primer, and distilled water (total reaction volume, 10 µL). The reaction was carried out with a 30-sec hot start at 96 °C, followed by 25 cycles of 10 s at 96 °C, 5 s at 50 °C, and 4 min at 60 °C, with a final elongation step at 60 °C for 4 min. The reaction solution was purified using Sephadex G-50 superfine columns (GE Healthcare, Chicago, IL, USA) and dried before adding 15 µL of Hi-Di formamide (Life Technologies). The DNA was denatured at 95 °C for 2 min, incubated on ice for at least 5 min, and subjected to capillary electrophoresis on an ABI PRISM 3130xl instrument (Life Technologies) to determine the DNA sequence (Table 2).

### Statistical analyses

Mann–Whitney *U* or chi-squared tests were used to compare the clinical information between the GMA and non-GMA groups. Before association analysis, each tag SNP was evaluated for Hardy–Weinberg Equilibrium (HWE) using the chi-squared test. Allele and genotype frequencies were compared in three genetic models (allele, dominant minor allele, and recessive minor allele) using chi-squared or Fisher’s exact tests. We performed multivariate logistic regression analysis on tag SNPs that were significantly associated with GMA progression in univariate analysis to verify whether their effects on age and genotype were independent.

Statistical analyses were performed using SNPAlyze software (version 7.0; Dynacom Co., Ltd., Yokohama, Japan) for the chi-squared and Fisher’s exact tests to compare frequencies of alleles or genotypes. Comparison of clinical information and multivariate logistic regression analyses were performed using IBM SPSS (version 20; IBM Japan, Tokyo, Japan) or Prism 5 (GraphPad Software, Inc., La Jolla, CA, USA), and odds ratios (OR) and 95% confidence intervals (95% CI) were calculated. Statistical significance was set at *p* < 0.05.

Genetic diagnosis was performed using gene polymorphisms that showed significant associations. The sensitivity, specificity, positive predictive value (PPV), and negative predictive value (NPV) of each polymorphism were calculated to evaluate the usefulness of the polymorphisms as biomarkers.

## Results

### Characteristics of the study subjects

Table 3 presents the characteristics of the GMA and non-GMA groups. There were no significant differences in sex between the two groups. However, the subjects in the GMA group were significantly older than those in the non-GMA group (*p* < 0.002).

**Table 3:** Clinical characteristics of *H. pylori*-positive subjects

Characteristics	GMA	non-GMA	P value*
Number of patients	94	106	-
Age, mean ± SD (years)	59.1 ± 9.51	54.8 ± 10.92	0.002
Gender(male/female)	37/57	50/56	0.266

*Characteristics were analyzed using Mann–Whitney U or chi-squared tests.

### Association between genetic polymorphisms and GMA susceptibility

We conducted HWE tests on all tag SNPs in the three candidate genes. Among the 14 SNPs, two SNPs, rs715948 in *LRP1* and rs9577502 in *LAMP1*, did not meet the HWE criteria and were excluded from further analyses. The other tag SNPs satisfied HWE (*p* > 0.05). The allelic, minor allele dominant, and minor allele recessive models were used for SNP analyses in this study.

Table 4 shows the distribution of the genotypes of each tag SNP between the GMA and non-GMA groups. The frequencies of recessive model rs1800137 (T/T genotype) (2.1% vs. 10.4%, *p* = 0.018, OR = 0.188) and rs11172113 (C/C genotype) (2.1% vs. 8.5%, *p* = 0.049, OR = 0.234) in *LRP1* were significantly lower in the GMA group than those in the non-GMA group. Inversely, the major allele dominant models of rs1800137 and rs11172113 in *LRP1* were 5.32 and 4.27 times more likely to cause GMA, respectively. The frequencies of the dominant model rs4423118 (G/A or A/A genotype) (61.7% vs. 46.3%, *p* = 0.029, OR = 1.874) and rs58618380 (T/A or A/A genotype) (62.8% vs. 47.2%, *p* = 0.027, OR = 1.888) in *CAPZA1* were higher in the GMA group than those in the non-GMA group.

**Table 4:** Frequencies of genotypes of tag SNPs in the GMA and non-GMA goups

Gene	SNP	Genotype	GMA	non-GMA	Genetic model	OR(95% CI)	*P* value*
			n = 94	n = 106			
*LRP1*	rs1799986	G/G	68 (72.3)	77 (72.6)	Allele model	1.107 (0.637–1.924)	0.720
		G/A	23 (24.5)	28 (26.4)	Dominant model	1.015 (0.545–1.891)	0.962
		A/A	3 (3.2)	1 (0.9)	Recessive model	3.462 (0.354–33.861)	0.257
	rs1800137	C/C	62 (66.0)	67 (63.2)	Allele model	0.715 (0.439–1.166)	0.178
		C/T	30 (31.9)	28 (26.4)	Dominant model	0.887 (0.496–1.586)	0.685
		T/T	2 (2.1)	11 (10.4)	Recessive model	0.188 (0.041–0.870)	0.018
	rs1800159	G/G	28 (29.8)	34 (32.1)	Allele model	0.995 (0.670–1.476)	0.979
		G/A	48 (51.1)	49 (46.2)	Dominant model	1.113 (0.610–2.031)	0.727
		A/A	18 (19.1)	23 (21.7)	Recessive model	0.855 (0.428–1.706)	0.656
	rs11172113	T/T	55 (58.5)	73 (68.9)	Allele model	1.129 (0.696–1.831)	0.623
		T/C	37 (39.4)	24 (22.6)	Dominant model	1.569 (0.878–2.804)	0.128
		C/C	2 (2.1)	9 (8.5)	Recessive model	0.234 (0.049–1.113)	0.049
	rs11837145	C/C	26 (27.7)	43 (40.6)	Allele model	1.138 (0.766–1.692)	0.523
		C/A	52 (55.3)	38 (35.8)	Dominant model	1.785 (0.984–3.238)	0.055
		A/A	16 (17.0)	25 (23.6)	Recessive model	0.665 (0.330–1.339)	0.251
*CAPZA1*	rs4423118	G/G	36 (38.3)	57 (53.8)	Allele model	1.341 (0.884–2.035)	0.168
		G/A	47 (50.0)	34 (32.1)	Dominant model	1.874 (1.066–3.296)	0.029
		A/A	11 (11.7)	15 (14.2)	Recessive model	0.804 (0.350–1.849)	0.607
	rs4525162	C/C	30 (31.9)	34 (32.1)	Allele model	0.947 (0.639–1.403)	0.785
		C/T	41 (43.6)	43 (40.6)	Dominant model	1.007 (0.556–1.827)	0.981
		T/T	23 (24.5)	29 (27.4)	Recessive model	0.860 (0.456–1.623)	0.642
	rs10885188	C/C	24 (25.5)	33 (31.1)	Allele model	1.021 (0.689–1.514)	0.917
		C/A	53 (56.4)	49 (46.2)	Dominant model	1.319 (0.710–2.450)	0.381
		A/A	17 (18.1)	24 (22.6)	Recessive model	0.754 (0.377–1.511)	0.426
	rs58618380	T/T	35 (37.2)	56 (52.8)	Allele model	1.254 (0.827-1.900)	0.287
		T/A	50 (53.2)	34 (32.1)	Dominant model	1.888 (1.072–3.325)	0.027
		A/A	9 (9.6)	16 (15.1)	Recessive model	0.596 (0.250–1.420)	0.239
	rs71481135	T/T	67 (71.3)	74 (69.8)	Allele model	0.928 (0.546–1.577)	0.783
		T/C	24 (25.5)	28 (26.4)	Dominant model	0.932 (0.507–1.714)	0.821
		C/C	3 (3.2)	4 (3.8)	Recessive model	1.000 (0.183–3.857)	0.823
*LAMP1*	rs9577230	T/T	74 (78.7)	73 (68.9)	Allele model	0.659 (0.376–1.157)	0.145
		T/C	17 (18.1)	29 (27.4)	Dominant model	0.598 (0.314–1.137)	0.115
		C/C	3 (3.2)	4 (3.8)	Recessive model	0.841 (0.183–3.857)	1.000
	rs12871648	A/A	41 (43.6)	39 (36.8)	Allele model	0.971 (0.645–1.462)	0.887
		A/C	39 (41.5)	57 (53.8)	Dominant model	0.753 (0.427–1.327)	0.326
		C/C	14 (14.9)	10 (9.4)	Recessive model	1.680 (0.708–3.986)	0.236

Genotype distribution and three genetic models, odds ratio, and p-value for each tag SNPs between the GMA and non-GMA groups are shown.

*Alleles and genotypes in the three genetic models were compared using chi-squared or Fisher’s exact tests (OR, odds ratio; CI, confidence interval).

Next, we conducted a multivariate logistic regression analysis to determine whether the C/C or C/T genotype at rs1800134 in *LRP1*, T/A or A/A genotype at rs58618380 in *CAPZA1*, and age were independently associated with GMA. Table 5 shows the OR (95% CI) and *p*-values obtained using the multivariate logistic regression analysis. A significant association with GMA risk was observed in the C/C or C/T genotype at rs1800137 in *LRP1* (OR = 5.181; 95% CI = 1.093–24.390; *p* = 0.038), T/A or A/A genotype at rs58618380 in *CAPZA1* (OR = 1.973; 95% CI = 1.100–3.549, *p* = 0.023), and age (OR = 2.264; 95% CI = 1.263–4.057; *p* = 0.006).

**Table 5:** Multivariate logistic regression analysis of rs1800137 in LRP1 and age

Factor	OR (95% CI)	*P* value
C/C or C/T genotype at rs1800137 in LRP1	5.181 (1.093–24.390)	0.038
T/A or A/A genotype at rs58618380 in CAPZA1	1.973 (1.100-3.549)	0.023
Age	2.264 (1.263–4.057)	0.006

Multivariate analysis revealed that C/C or C/T genotype at rs1800137 in LRP1 and T/A or A/A genotype at rs58618380 in CAPZA1, and age are independently associated with the GMA

### Association between genetic polymorphisms and endoscopic GMA (EGMA) susceptibility

There were five missing datasets in the EGMA classification for the 200 participants. Therefore, 195 EGMA datasets were available. Table 6 shows the relationship between the presence of EGMA and genotypes at rs1800137 in *LRP1* and rs58618380 in *CAPZA1*. Unfortunately, none of the SNPs reached statistical significance.

**Table 6:** Frequencies of genotypes of tag SNPs in the EGMA and non-EGMA goups

SNP ID	Genotype	EGMA	non-EGMA	Genetic model	OR(95%CI)	P value
		n = 123	n = 72			
rs1800137	C/C	83	44	Allele model	1.489	0.115
		(67.5)	(61.1)		(0.906–2.447)	
	C/T	35	20	Dominant model	1.321	0.368
		(28.5)	(27.8)		(0.721–2.420)	
	T/T	5	8	Recessive model	2.950	0.057
		(4.1)	(11.1)		(0.927–9.392)	
rs58618380	T/T	59	30	Allele model	1.382	0.141
		(48.0)	(41.7)		(0.898–2.123)	
	T/A	52	29	Dominant model	1.291	0.368
		(42.3)	(40.3)		(0.718–2.322)	
	A/A	12	13	Recessive model	2.038	0.094
		(9.8)	(18.1)		(0.875–4.749)	

Genotype distribution and three genetic models, odds ratio, and p-value for each tag SNPs between the EGMA and non-EGMA groups are shown.

*Alleles and genotypes in the three genetic models were compared using chi-squared or Fisher’s exact tests (OR, odds ratio; CI, confidence interval).

### Verification of polymorphisms as useful biomarkers

The C/C or C/T genotype rs1800137 of *LRP1*, which showed an independent correlation with GMA, was used as a biomarker for genetic diagnosis. The sensitivity, specificity, PPV, and NPV were also calculated (Table 7).

**Table 7:** The sensitivity, specificity, positive predictive value, and negative predictive value of the C/C or C/T genotype of rs1800137 in LRP1 and/or the T/A or A/A genotype of rs58618380 in CAPZA1 as a biomarker of GMA progression

Biomarker	*LRP1*		*CAPZA1*		Statistical results			Genetic diagnosis			
	rs1800137		rs58618380		OR (95% CI)	P value*		sensitivity	specificity	PPV	NPV
Biomarker 1	C/C or C/T		-		5.326 (1.149–24.70)	0.021		97.9	10.4	49.2	84.6
Biomarker 2	-		T/A or A/A		1.888 (1.030–1.923)	0.033		62.8	52.8	54.1	61.5
Biomarker 3	C/C or C/T		T/A or A/A		2.270 (1.287–4.005)	0.005		61.7	58.5	56.9	63.3

*Factors were statistically analyzed using chi-squared tests.

## Discussion

To the best of our knowledge, the present study is the first to suggest that the C/C or C/T genotype at rs1800137 in *LRP1* and T/A or A/A genotype at rs58618380 in *CAPZA1* are associated with the development of GMA. Multivariate analysis revealed that the C/C or C/T genotype at rs1800137, T/A or A/A genotype at rs58618380, and age were independently associated with GMA. However, the genotypes of rs1800137 in *LRP1* and rs58618380 in *CAPZA1* did not reach statistical significance with the endoscopic classification.

Chronic *H. pylori* infection is the leading cause of GMA. *H. pylori* injects CagA into gastric epithelial cells via a bacterial Type IV secretion system. CagA variants are classified into East Asian- and Western-type variants. East Asian CagA is more toxic than Western CagA and strongly promotes gastric cancer development [26]. These two CagA types differ in their binding affinity for Src homology 2 (SHP2) and protein tyrosine phosphatase [27] and induce the cytokines IL-6 [28] and IL-8 [29]. These two cytokines increase inflammation in gastric epithelial cells [30] and play an essential role in GMA. CagA is degraded by autophagy following its translocation [19]. The half-life of CagA in gastric epithelial cells is approximately 200 min [31]. VacA, another pathogenic factor of *H. pylori*, is classified into two types, m1 and m2, based on differences in its gene sequence. Almost all (84/87) Japanese *H. pylori*-positive patients have the m1-type VacA [32]. m1-type VacA binds to *LRP1*, whereas m2-type VacA does not [33]. Once VacA binds to *LRP1*, the *LRP1*–ICD complex moves into the nucleus, increases *LAMP1* expression, and induces autophagy. In contrast, *CAPZA1* suppresses *LAMP1* expression by binding to the *LAMP1* proximal promoter, which is necessary for autolysosome function, and negatively regulates autophagy [20, 34]. Therefore, *LRP1*, *LAMP1*, and *CAPZA1* are associated with the accumulation of translocated CagA and may be important for the progression of GMA.

**Fig. 3:**
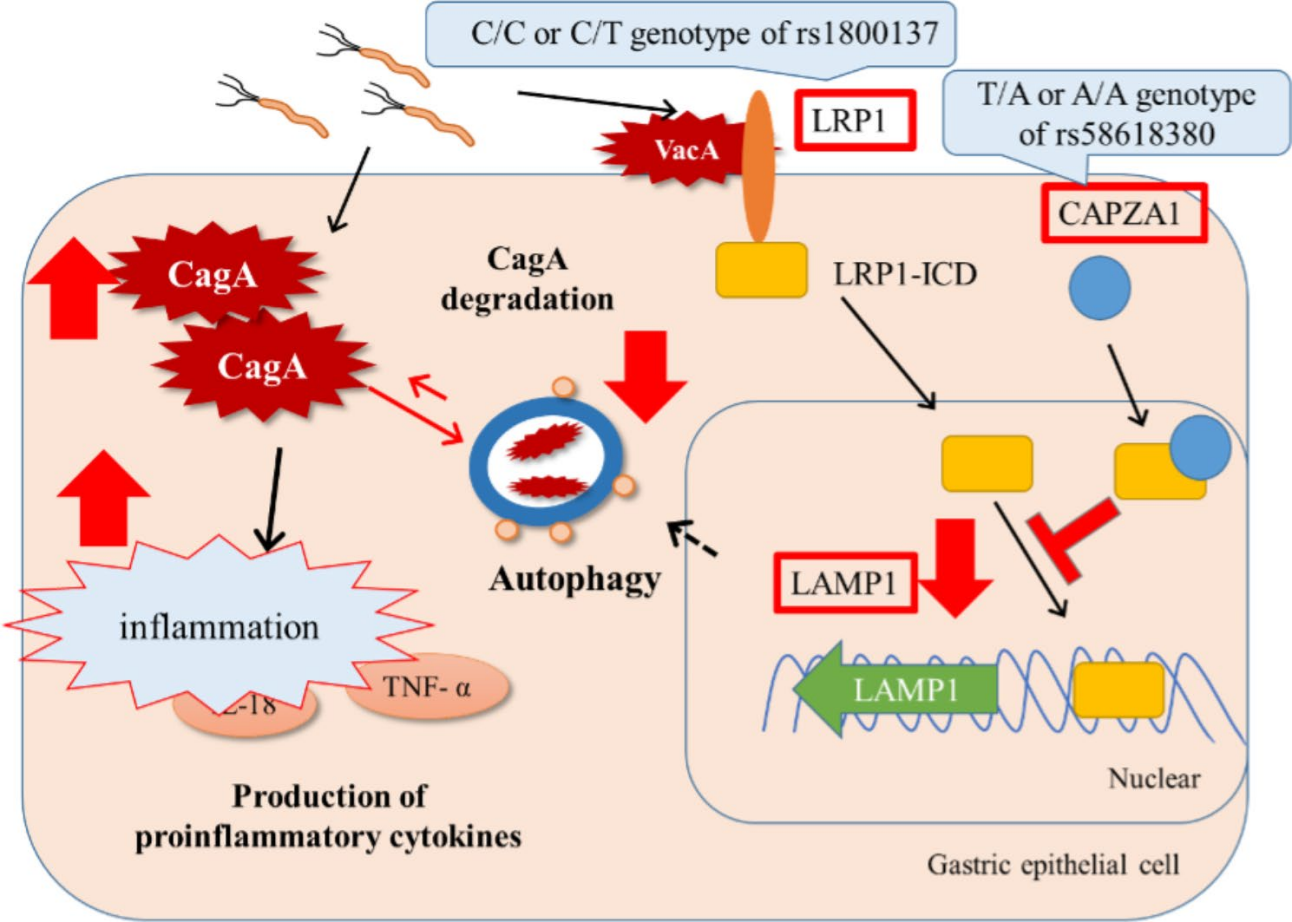
Putative effects of *LRP1* and *CAPZA1* polymorphism on host defenses against CagA protein in gastric epithelial cells. The C/C or C/T genotype of rs1800137 in *LRP1* or T/A or A/A genotype of rs58618380 in *CAPZA1* may decrease *LAMP1* expression, reduce autophagy, and accelerate CagA-mediated inflammation. Abbreviations: *LAMP1*, lysosome-associated membrane protein 1; *CAPZA1*, capping actin protein of muscle Z-line alpha subunit 1; CagA, cytotoxin-associated gene A; *LRP1*, low-density lipoprotein receptor-related protein 1; *LRP1*-ICD, LRP1 intracellular domain; IL-1β, interleukin 1 beta; IL-18, interleukin 18; TNFα, tumor necrosis factor-alpha

Although functional analyses of rs1800137 in *LRP1* and rs5861830 in *CAPZA1* were not performed in this study, we speculate that the molecular mechanism underlying GMA development in the case of the C/C or C/T genotype of *LRP1* at rs1800137 and T/A or A/A genotype of *CAPZA1* at rs58618380 may be due to decreased autophagy. Reduced autophagy suppressed CagA degradation, leading to intracellular CagA accumulation, increased pro-inflammatory cytokine production, persistent inflammation, and the development of GMA (Fig. 3). We could not find any reports of rs58618380 in *CAPZA1*. rs58618380 is located on chr10:111456283, where an intron is located. T/A or A/A genotypes at rs58618380 could enhance the expression of *CAPZA1*. When *H. pylori* is infected into *CAPZA1*-overexpressing epithelial gastric cells, cluster-of-differentiation gene 44 variant isoform 9 (CD44v9)-positive cells and cell-surface markers associated with cancer stem cells are induced [34]. In gastric cancer, CD44v9 regulates reactive oxygen species, resulting in subsequent therapeutic resistance, recurrence, and metastasis of tumors [35]. The CD44v9-positive group has a higher recurrence rate than the CD44v9-negative group [35]. Although CD44v9 emerges in response to injury and contributes to the gastric epithelium [36], there are no reports of CD44v9 directly causing GMA. Further functional studies of rs58618380 in *CAPZA1* and CD44v9 are required.

However, there are only two reports on *LRP1* mutations in gastric cancer. Nakamura et al. reported that *LRP1* mRNA levels in gastric cancer with *LRP1* mutations were significantly lower than those in gastric cancer without *LRP1* mutations. Furthermore, CagA accumulation is significantly increased in gastric cancer tissues with *LRP1* mutations [21]. Polymorphisms of *LRP1* may be associated with CagA accumulation and atrophic gastritis. Another study reported that rs1800137 is in exon 8, which is an exon-splicing enhancer, and the T/T genotype of rs1800137 might decrease *LRP1* expression [37]. This result contrasts with ours; however, these studies have not been conducted on atrophic gastritis. Further functional analyses of *LRP1* are required to elucidate the relationship between autophagy and GMA during *H. pylori* infection.

We tested the usefulness of SNPs in *LRP1* and *CAPZA1* as biomarkers for predicting GMA progression. The C/C or C/T genotype at rs1800137 of *LRP1*, T/A or A/A genotype at rs58618380 in *CAPZA1*, and combination of both SNPs do not have enough power to be used as biomarkers. However, the autophagy-related genes *LRP1* and *CAPZA1* contribute to *H. pylori* infection-induced GMA, suggesting that there may be molecules that could serve as novel drug targets. These molecules can delay the onset and progression of GMA from persistent infections and eradicate *H. pylori* by degrading CagA.

This study had several limitations. First, the sample size was small. Further prospective studies with larger sample sizes for exploratory studies are required to confirm the association between *LRP1* and *CAPZA1* SNPs and GMA development. Second, functional analysis of *LRP1* and *CAPZA1* polymorphisms in CagA and GMA was not performed. Moreover, no follow-up surveys were conducted on the enrolled subjects. In addition, we did not examine *H. pylori* staining and bacterial virulence factors, such as neutrophil-activating protein (Nap), duodenal ulcer-promoting gene A (dupA), outer inflammatory protein (OpiA), and lipopolysaccharides. These factors are involved in the progression of inflammation and tissue damage [38]. Thus, these factors may be involved in the progression of GMA. We hope to conduct future exploratory studies using propensity score matching to match these bacterial virulence factors.

Nevertheless, such genetic testing may provide future directions for precision medicine for individuals who are more likely to develop GMA.

## Conclusions

In this study, we identified *LRP1* and *CAPZA1* as susceptibility genes for GMA with *H. pylori* infection. Because GMA progression is associated with the development of gastric cancer, it is important to encourage *H. pylori*-infected individuals with these two SNPs to undergo timely endoscopic testing for early-stage gastric cancer.

Further investigations of *LRP1* and *CAPZA1* polymorphisms and their functions are required to elucidate the pathophysiological differences between GMA and gastric cancer. Pathophysiological findings and the establishment of reliable biomarkers leading to the development of new therapeutic GMA drugs that degrade CagA to reduce gastric cancer risk are required.

## Data Availability

The datasets generated during the current study are not publicly available due to that is not written in informed consent.
